# Ideal type-II Weyl points in topological circuits

**DOI:** 10.1093/nsr/nwaa192

**Published:** 2020-08-28

**Authors:** Rujiang Li, Bo Lv, Huibin Tao, Jinhui Shi, Yidong Chong, Baile Zhang, Hongsheng Chen

**Affiliations:** Interdisciplinary Center for Quantum Information, State Key Laboratory of Modern Optical Instrumentation, College of Information Science and Electronic Engineering, Zhejiang University, Hangzhou 310027, China; ZJU-Hangzhou Global Science and Technology Innovation Center, Key Laboratory of Advanced Micro/Nano Electronic Devices & Smart Systems of Zhejiang, Zhejiang University, Hangzhou 310027, China; Key Laboratory of In-Fiber Integrated Optics of Ministry of Education, College of Physics and Optoelectronic Engineering, Harbin Engineering University, Harbin 150001, China; School of Software Engineering, Xi’an Jiaotong University, Xi’an 710054, China; Key Laboratory of In-Fiber Integrated Optics of Ministry of Education, College of Physics and Optoelectronic Engineering, Harbin Engineering University, Harbin 150001, China; Division of Physics and Applied Physics, School of Physical and Mathematical Sciences, Nanyang Technological University, Singapore 637371, Singapore; Centre for Disruptive Photonic Technologies, Nanyang Technological University, Singapore 637371, Singapore; Division of Physics and Applied Physics, School of Physical and Mathematical Sciences, Nanyang Technological University, Singapore 637371, Singapore; Centre for Disruptive Photonic Technologies, Nanyang Technological University, Singapore 637371, Singapore; Interdisciplinary Center for Quantum Information, State Key Laboratory of Modern Optical Instrumentation, College of Information Science and Electronic Engineering, Zhejiang University, Hangzhou 310027, China; ZJU-Hangzhou Global Science and Technology Innovation Center, Key Laboratory of Advanced Micro/Nano Electronic Devices & Smart Systems of Zhejiang, Zhejiang University, Hangzhou 310027, China

**Keywords:** topological circuits, Weyl points, surface states, band structure

## Abstract

Weyl points (WPs), nodal degenerate points in three-dimensional (3D) momentum space, are said to be ‘ideal’ if they are symmetry-related and well-separated, and reside at the same energy and far from nontopological bands. Although type-II WPs have unique spectral characteristics compared with type-I counterparts, ideal type-II WPs have not yet been reported because of a lack of an experimental platform with enough flexibility to produce strongly tilted dispersion bands. Here, we experimentally realize a topological circuit that hosts only topological bands with a minimal number of four ideal type-II WPs. By stacking two-dimensional (2D) layers of inductor-capacitor (*LC*) resonator dimers with the broken parity inversion symmetry (*P*), we achieve a strongly tilted band structure with two group velocities in the same direction, and topological surface states in an incomplete bandgap. Our results establish an ideal system for the further study of Weyl physics and other exotic topological phenomena.

## Teaser

The unique gadolinium MOF based nanosheets with the paddlewheel frameworks have been constructed to realize the ultrahigh drug loading and bimodal-imaging guided cancer chemotherapy.

## INTRODUCTION

Weyl points (WPs) are nodal degenerate points where two linear energy spectra intersect in 3D momentum space [1]. In a 3D crystal, if all the WPs are symmetry-related and well-separated, and reside at the same energy and far from nontopological bands, the crystal is called an ‘ideal’ Weyl system [2–4]. In an ideal Weyl system, it is relatively straightforward to distinguish WPs from other types of band crossings and to observe the related phenomena such as topological surface states [5]. Moreover, ideal WPs are particularly useful in some realistic and innovative device applications [6]. To date, ideal type-I WPs with symmetric cone spectra have been observed in semimetals [7], and also in artificial photonic crystals that utilize the flexibility and diversity of synthetic classical structures [4].

One class of WPs, known as type-II WPs, have never previously been observed in an ideal form, although, conceptually, type-II WPs can be transited from type-I counterparts by increasing the kinetic component in the general Weyl Hamiltonian [8]. In contrast to the well-studied type-I WPs [9–16], it was not until 2015 that type-II WPs were theoretically proposed [17,18] and then experimentally confirmed in condensed matter [19–21], photonic [22–24] and acoustic systems [25]. Type-II WPs exhibit unique characteristics that include strongly tilted cone spectra, two group velocities in the same direction and topological surface states in an incomplete bandgap [18,26]. Moreover, type-II WPs can be used to model some fascinating phenomena of relevance to astrophysics such as Hawking radiation and gravitational lensing [27,28]. Unfortunately, the realization of ideal type-II WPs has been hindered by the lack of an experimental platform with enough flexibility to produce strongly tilted dispersion bands [8]. To the best of our knowledge, ideal type-II WPs have only been theoretically predicted in certain condensed matter systems without experimental validation [3,29,30].

Here, we report on the realization of a minimal number of four ideal type-II WPs in a topological circuit that reside at the same energy with a large momentum separation. Using site-resolved transmission measurement and mapping out the band structures, we confirm the existence of two key signatures of type-II WPs: (i) a strongly tilted band structure with two group velocities in the same direction, and (ii) the existence of topological surface states in an incomplete bandgap. Unlike previous proposals [3,29,30], our design utilizes a macroscopic circuit system. In this experimental platform, lattice sites can be wired in an arbitrary manner with arbitrary numbers of connections per node and long-range connections, and the hopping strengths are independent of the distance between the nodes [6]. This flexible and highly customizable connectivity, and the distance independent hopping allow easy fabrication of a circuit lattice with two topological bands and no nontopological bands. Under broken *P* and preserved time reversal symmetry (*T*) conditions, we obtain the minimal number of four ideal type-II WPs that reside at the same energy with a large momentum separation.

## RESULTS

### Design of the topological circuit

**Figure 1.**
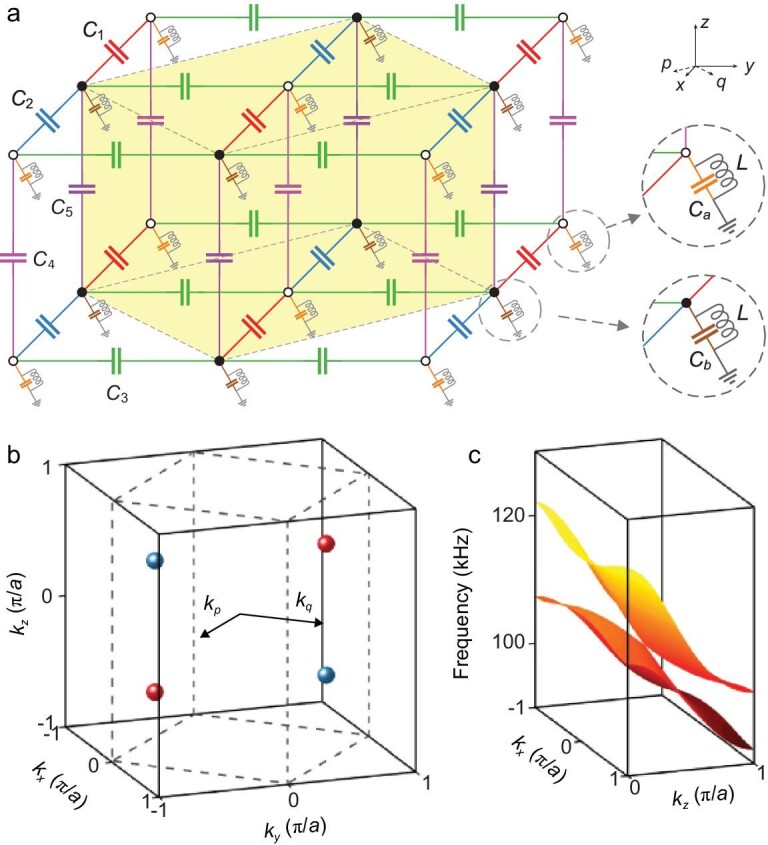
Topological circuit with ideal type-II WPs. (a) Schematic of the circuit. The white and black nodes are wired to grounded parallel *LC* resonators with two different resonant frequencies. Nearest-neighbor nodes are wired by coupling capacitors in the *x, y* and *z* directions with capacitances *C*_1_ = 18 pF, *C*_2_ = 82 pF, *C*_3_ = 100 pF, *C*_4_ = 270 pF and *C*_5_ = 120 pF. The grounded capacitances are *C_a_* = 1.5 nF and *C_b_*_ _= 1.8 nF, and the inductances are *L* = 1 mH. The yellow cube denotes a unit cell. The *p* and *q* directions are defined as $p = {{( {x - y} )} / {\sqrt 2 }}$ and $q = {{( {x + y} )} /{\sqrt 2 }}$, respectively. (b) Schematic of momentum space, showing the 3D Brillouin zone (dashed lines) and the four ideal type-II WPs at (0, ±2/3, ±1/2) π/*a*, where *a* is the spacing between nearest-neighbor resonator nodes. The WPs occur at energy 104.0 kHz and form pairs with opposite chirality, with each point acting as either a source (red spheres) or sink (blue spheres) of Berry curvature. The *k_p_* and *k_q_* directions are defined as ${k_p} = {{( {{k_x} - {k_y}} )} /{\sqrt 2 }}$ and ${k_q} = {{( {{k_x} + {k_y}} )} /{\sqrt 2 }}$, respectively. (c) A strongly tilted 2D band structure for the circuit in the *k_x_*−*k_z_* plane with *k_y_* = ±2π/3*a*.

Our topological circuit exhibiting ideal type-II WPs is constructed by stacking 2D layers of *LC* resonator dimers with broken *P*. As depicted in Fig. 1a, the white and black nodes are wired to two different types of grounded parallel *LC* resonators, and the nearest-neighbor nodes are wired by coupling capacitors in the *x, y* and *z* directions. The inductors in all resonators are identical. For an isolated layer in the *x*−*y* plane with identical grounded capacitors *C_a_* = *C_b_* and identical coupling capacitors *C*_1_ = *C*_2_ = *C*_3_, there are two bands that are degenerate at the boundary of the square 2D Brillouin zone (BZ) and exhibit a quadratic degeneracy at the corners [31]. By stacking identical layers along the *z* direction with *C*_4_ = *C*_5_, the band degeneracy points (BDPs) form a square tube with rotational axis along the *k_z_* direction (see Supplementary data sections 1 and 2). To break *P*, we break the mirror symmetry in the *x* direction *M_x_* := *x* → −*x* by setting *C*_1_ ≠ *C*_2_, which leads to the splitting of the square degeneracy in the *k_x_*−*k_y_* plane [26,31]. In the 3D BZ, the BDPs form a pair of lines along the *k_z_* direction with *k_x_* = 0 and *k_y_* being determined by the sum of *C*_1_ and *C*_2_, and they have linear dispersion in the *k_x_* and *k_y_* directions. Here, we set *C*_1_ + *C*_2_ = *C*_3_, so that the degenerate lines are projected to (0, ±2/3)π/*a*, where *a* is the spacing between the nearest resonator nodes. To isolate the BDPs, we break the line degeneracy by setting *C*_4_ ≠ *C*_5_. This produces two BDPs at (0, ±2/3, 0)π/*a* with quadratic dispersion in the *k_z_* direction. To move the BDPs to the positions that have linear dispersion, we use resonators with two different resonant frequencies by setting *C_a_* ≠ *C_b_* and, specifically, we take *C_a_* – *C_b_* = −2(*C*_4_ – *C*_5_). Then the original two BDPs split into four points at (0, ±2/3, ±1/2)π/*a*, which are the type-II WPs.

Under broken *P* and preserved *T* conditions, the minimum number of type-II WPs is four [6]. As shown in Fig. 1b, the four type-II WPs are distributed in momentum space with a large separation, where the first BZ is indicated by dashed lines. Type-II WPs exist in pairs with opposite chirality and each point acts as either a source (red spheres) or sink (blue spheres) of Berry curvature (see Supplementary data section 3). This topological circuit has two mirror symmetries *M_y_* := *y* → −*y* and *M_z_* := *z* → −*z*, and *T*. Considering that mirror symmetry requires a WP located at **k** to create another WP at −**k** with the opposite chirality, and *T* requires another WP at −**k** to have the same chirality [6], the four type-II WPs satisfy the constraints imposed by the symmetries; i.e. they are symmetry-related. Notably, all these type-II WPs reside at the same energy 104.0 kHz under the realistic circuit parameters mentioned in the caption of Fig. 1. The capacitive coupling from the identical resonators in the *z* direction leads to a strongly tilted band structure in the *k_x_*−*k_z_* plane with *k_y_* = ±2π/3*a*, as shown in Fig. 1c. It can thus be concluded that this topological circuit is an ideal type-II Weyl system with no nontopological bands. The minimal number of four type-II WPs are symmetry-related and well-separated, and reside at the same energy.

### Observation of ideal type-II WPs

To verify the existence of ideal type-II WPs, we experimentally implement two topological circuits using stacked circuit boards, where one is periodic along the *x, y* and *z* directions, and the other one is finite along the $p = {{( {x - y} )} /{\sqrt 2 }}$ direction and periodic along the $q = {{( {x + y} )} / {\sqrt 2 }}$ and *z* directions (see Methods, Circuit fabrication). As a periodic boundary can mimic an infinite structure [32], the two topological circuits are equivalent to two different theoretical lattices, one infinite along all three directions and the other finite along the *p* direction and infinite along the other directions. We excite and probe the resonator nodes of topological circuits using a network analyzer. Band structures in different directions of the BZ are mapped out by applying a Fourier transform to the complex transmission coefficients in real space to validate the existence of ideal type-II WPs (see Methods, Measurement setup and band structure reconstruction, and Supplementary data section 4).

**Figure 2.**
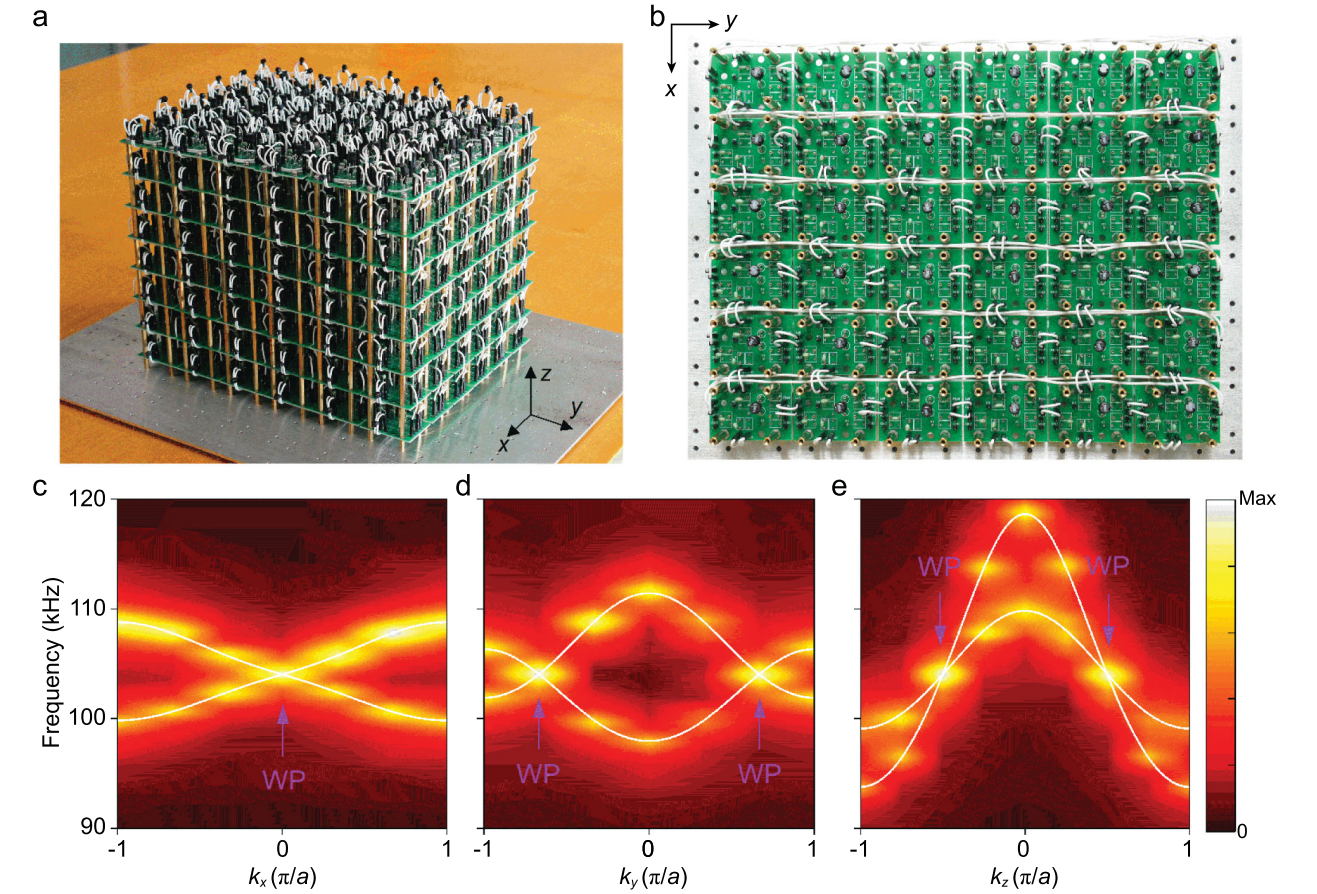
Observation of a strongly tilted band structure. (a and b) Photographs of the (a) 3D structure and (b) bottom layer for a topological circuit with periodic boundary conditions along the *x, y* and *z* directions. (c–e) Experimentally measured 1D band structures obtained by sweeping *k_x_* at (*k_y_, k_z_*) = (±2/3, ±1/2)π/*a, k_y_* at (*k_x_, k_z_*) = (0, ±1/2)π/*a*, and *k_z_* at (*k_x_, k_y_*) = (0, ±2/3)π/*a*, respectively. The curves correspond to theoretical results. Four ideal type-II WPs are located at (0, ±2/3, ±1/2)π/*a* with the same energy 104.0 kHz. In (e), the two strongly tilted bands imply that the group velocities are in the same direction, which is a signature of type-II WPs.

The key physical signature of type-II WPs is a strongly tilted band structure with two group velocities in the same direction. Figure 2a and b shows the fabricated topological circuit with periodic boundaries along all three directions. Experimentally, bulk states are excited and probed by site-resolved measurements. Various one-dimensional (1D) slices of the measured 3D band structures are shown in Fig. 2c–e. The corresponding theoretical bands calculated from the equivalent infinite lattice are also included in the plots. Sweeping *k_x_* at (*k_y_, k_z_*) = (±2/3, ±1/2)π/*a*, one degenerate point is observed at *k_x_* = 0 (Fig. 2c). Similarly, sweeping *k_y_* at (*k_x_, k_z_*) = (0, ±1/2)π/*a* reveals two degenerate points at *k_y_* = ±2π/3*a* (Fig. 2d), and sweeping *k_z_* at (*k_x_, k_y_*) = (0, ±2/3)π/*a* shows two degenerate points at *k_z_* = ±π/2*a* (Fig. 2e). The strongly tilted bands near each degeneracy imply that the two group velocities are in the same direction, which is a signature of type-II WPs. According to the **k · p** model, the band structures have linear dispersion at these ideal type-II WPs (see Supplementary data section 5).

**Figure 3.**
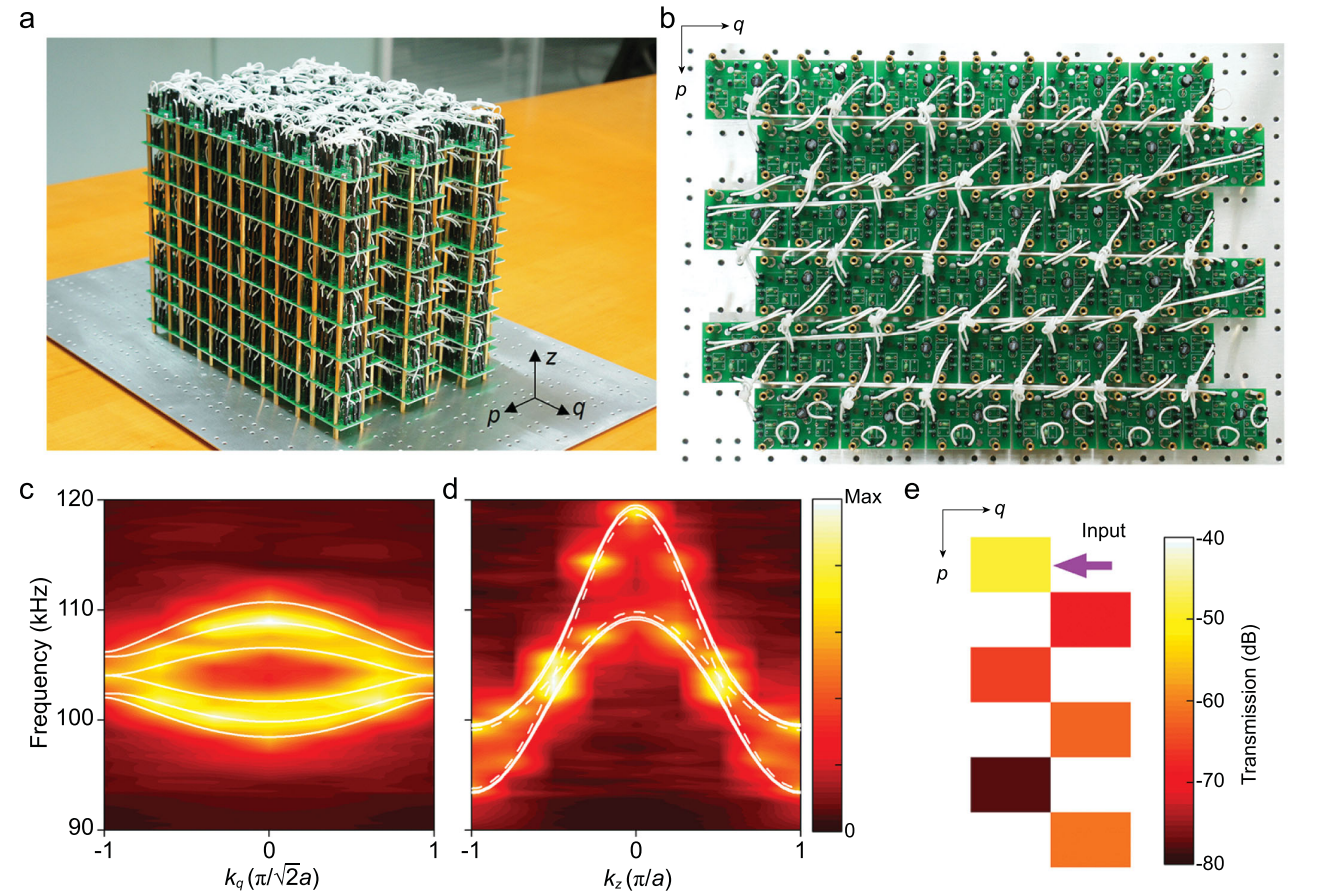
Observation of topological surface states in an incomplete bandgap. (a and b) Photograph of the (a) 3D structure and (b) bottom layer for a topological circuit that is finite along the *p* direction and periodic along the *q* and *z* directions. (c and d) Experimentally measured band structures along the *k_q_* direction with *k_z_* = ±π/2*a* and along the *k_z_* direction with ${k_q} = \pm {\pi / {\sqrt 2 a}}$, respectively. The curves correspond to theoretical results. In (d), the dashed curves correspond to two surface states, and the solid curves correspond to bulk states. (e) Transmission distribution of the topological surface states with (*k_q_, k_z_*) = ($ \pm {\pi / {\sqrt 2 a}}$, ±π/2*a*) along the *p* direction at 104.0 kHz, where the arrow indicates the input port. The existence of topological surface states in an incomplete bandgap is another signature of type-II WPs.

Another exciting signature of type-II WPs is the existence of topological surface states in an incomplete bandgap. As shown in Fig. 3a and b, we fabricate the topological circuit with a finite boundary along the *p* direction and periodic boundaries along the other directions. To observe surface states, we excite the resonator nodes from the two opposite boundaries normal to the *p* direction. The experimental band structures are shown in Fig. 3c and d, where ${k_q} = {{( {{k_x} + {k_y}} )} /{\sqrt 2 }}$. Similarly, the corresponding theoretical band structures calculated from the equivalent circuit lattice (which is finite along the *p* direction and infinite along the other directions) are shown by the curves. Because of the finite boundary, there are multiple dispersion bands in Fig. 3c, where *k_z_* = ±π/2*a*. When the momentum *k_q_* satisfies $| {{k_q}} | \ge {{\sqrt 2 \pi } /{3a}}$, the dispersion bands create an incomplete bulk bandgap with two surface states [26]. As shown in Fig. 3d, when ${k_q} = \pm {\pi /{\sqrt 2 a}}$, an incomplete bandgap is formed by the solid curves that correspond to the bulk states, and the dashed curves that correspond to the surface states reside in the bandgap. The experimental bands at *k_z_* = ±π/2*a* are slightly overlapped because of the lossy effect from the serial resistance of inductors (see Supplementary data section 6). In contrast to the type-I WPs, the group velocities of the surface states for type-II WPs have the same sign for a fixed *k_z_* [26]. To validate the existence of topological surface states, in Fig. 3e we show the transmission distribution along the *p* direction at 104.0 kHz, where the arrow indicates the input port. The highly localized transmission distribution at the two opposite boundaries with a decay length $\xi < \sqrt 2 a$ (the distance between neighbor nodes in the *p* direction) validates the existence of the topological surface states with (*k_q_, k_z_*) = ($ \pm {\pi / {\sqrt 2 a}}$, ±π/2*a*). Here, bidirectional propagation of the surface states is experimentally observed because both the states with positive and negative *k_z_* are excited (see Supplementary data section 7). Besides, from the equifrequency contour at 104.0 kHz, we show the Fermi arcs connecting the projections of type-II WPs (see Supplementary data section 8).

## DISCUSSION

Based on the two topological circuits, we have demonstrated a strongly tilted band structure with two group velocities in the same direction and topological surface states in an incomplete bandgap. The two signatures validate the existence of a minimum number of four ideal type-II WPs that reside at the same energy with a large momentum separation and imply that our circuit system is an ideal type-II Weyl system. Compared with other 3D systems, periodic boundaries are easier to implement in circuit systems, which makes it feasible to construct band structures using compact circuits [33]. The properties of the circuit system such as Weyl frequency and group velocities are adjustable (see Supplementary data section 9). These advantages imply that our topological circuits provide a clean and adjustable platform to observe ideal type-II WPs.

## CONCLUSION

The experimental platform opens the door to the realization of new topological phases as well as possible practical device applications, such as analog signal processors. The flexibility and controllability of lumped element circuits are particularly beneficial for the realization of chiral anomaly in inhomogeneous Weyl materials [34], as well as the nonlinear [35–37], non-Hermitian [38–40], higher-order [37,41–44] and higher-dimensional [13,45,46] topological systems. Although our demonstration was carried out between 80 and 130 kHz, the design principle is generalizable to other frequency regimes such as microwave [47] and optical frequencies [48]. Furthermore, the building blocks of our circuit, *LC* resonators, can be regarded as atoms of a periodic lattice, i.e. a crystal, and the capacitive coupling corresponds to the bonding between these atoms. A similar lattice design may be applied to other classical systems such as photonic and acoustic systems to observe ideal type-II WPs.

## METHODS

### Circuit fabrication

We use the following set of circuit elements: (1) *L* = 1 mH (±1% tolerance, *Q* ∼ 75 @100 kHz) and (2) *C_a_* = 1.5 nF, *C_b_* = 1.8 nF, *C*_1_ = 18 pF, *C*_2_ = 82 pF, *C*_3_ = 100 pF, *C*_4_ = 270 pF and *C*_5_ = 120 pF (±1% tolerance). The tolerance values are achieved by delicately selecting the components using an Impedance Analyzer 4192A LF. Under these parameters, the frequency range of the topological circuits is 80–130 kHz.

### Measurement setup and band structure reconstruction

Network Analyzer HP 4195A was employed to measure the transmission coefficient between two resonator nodes of the topological circuits, where one port serves as the excitation and another port probes the response. Under an excitation, the eigenstates (bulk or surface states) of the topological circuit are excited. After probing the transmission spectra at all the resonator nodes with the frequency step 200 Hz, the complex transmission coefficients in real space are transformed to **k** space by applying a Fourier transform. To excite all the eigenstates, the white and black nodes at the boundaries are excited successively. The final band structure is the average of the results from the white and black nodes.

## Supplementary Material

nwaa192_Supplemental_File
