# Supplementary material for: Ideal type-II Weyl points in topological circuits
Source: Natl Sci Rev. 2020 Aug 28;8(7):nwaa192. doi: 10.1093/nsr/nwaa192 (PMC8310763; doi:10.1093/nsr/nwaa192)
Supplement: nwaa192_Supplemental_File [file nwaa192_supplemental_file.pdf]

Supplementary Information for "Ideal type-II Weyl points in topological circuits"

1. Hamiltonian of the topological circuit

The general Hamiltonian describing a type-II WP is $H(\mathbf{k}) = k_i A_{ij} \sigma_j$ ($i, j \in \{0, 1, 2, 3\}$ and repeated indices summed), where $\mathbf{k}$ is the wave vector, A is a coefficient matrix, and σ_j are the unitary matrix with $j = 0$ and three Pauli matrices with $j = 1, 2, 3$ [1]. The energy is $E_\pm(\mathbf{k}) = T(\mathbf{k}) \pm U(\mathbf{k})$, where $T(\mathbf{k})$ and $U(\mathbf{k})$ are the kinetic and potential components, respectively. The kinetic component which is linearly dependent on $\mathbf{k}$ tilts the Weyl cone. For a type-II WP, $T(\mathbf{k})$ is dominant over $U(\mathbf{k})$ for a particular direction.

For the topological circuit shown in Fig. 1**a**, according to Kirchhoff's current law, the voltages at the white and black nodes satisfy

$$\begin{aligned}
&LC_1 \frac{d^2}{dt^2} V(x-a,y,z) + LC_2 \frac{d^2}{dt^2} V(x+a,y,z) \\
&+LC_3 \frac{d^2}{dt^2} V(x,y-a,z) + LC_3 \frac{d^2}{dt^2} V(x,y+a,z) \\
&+LC_4 \frac{d^2}{dt^2} V(x,y,z-a) + LC_4 \frac{d^2}{dt^2} V(x,y,z+a) \\
&-L(C_a + C_1 + C_2 + 2C_3 + 2C_4) \frac{d^2}{dt^2} V(x,y,z) \\
&= V(x,y,z),
\end{aligned} \tag{S1}$$

and

$$\begin{aligned}
&LC_2 \frac{d^2}{dt^2} V(x-a,y,z) + LC_1 \frac{d^2}{dt^2} V(x+a,y,z) \\
&+LC_3 \frac{d^2}{dt^2} V(x,y-a,z) + LC_3 \frac{d^2}{dt^2} V(x,y+a,z) \\
&+LC_5 \frac{d^2}{dt^2} V(x,y,z-a) + LC_5 \frac{d^2}{dt^2} V(x,y,z+a) \\
&-L(C_b + C_1 + C_2 + 2C_3 + 2C_5) \frac{d^2}{dt^2} V(x,y,z) \\
&= V(x,y,z),
\end{aligned} \tag{S2}$$

respectively, where $C_a = C + \delta C$ and $C_b = C - \delta C$ are the grounded capacitors in the

inductor-capacitor (LC) resonators, and a is the spacing between the nearest-neighbor resonator nodes [2-5]. In the frequency domain, the above equations change to the following normalized forms

$$\begin{aligned}&\kappa_x V(x-a,y,z)+\nu_x V(x+a,y,z)+\kappa_y V(x,y-a,z)+\kappa_y V(x,y+a,z)\\&+\kappa_z V(x,y,z-a)+\kappa_z V(x,y,z+a)-(\delta_C+\kappa_z-\nu_z)V(x,y,z)\\&=\varpi V(x,y,z),\end{aligned} \tag{S3}$$

$$\begin{aligned}&\nu_x V(x-a,y,z)+\kappa_x V(x+a,y,z)+\kappa_y V(x,y-a,z)+\kappa_y V(x,y+a,z)\\&+\nu_z V(x,y,z-a)+\nu_z V(x,y,z+a)+(\delta_C+\kappa_z-\nu_z)V(x,y,z)\\&=\varpi V(x,y,z),\end{aligned} \tag{S4}$$

where $\kappa_x = C_1/C$ and $\nu_x = C_2/C$ are the hopping strengths in the x direction, $\kappa_y = C_3/C$ is the hopping strength in the y direction, $\kappa_z = C_4/C$ and $\nu_z = C_5/C$ are the hopping strengths in the z direction, $\delta_C = \delta C/C$, and

$$\varpi = 1+\kappa_x+\nu_x+2\kappa_y+\kappa_z+\nu_z-\frac{\omega_0^2}{\omega^2} \tag{S5}$$

is the normalized frequency with $\omega_0^2 = 1/LC$.

For a topological circuit with periodic boundaries, we assume the Bloch solutions $V(x,y,z) = \psi_{a,b} \exp(ik_x x + ik_y y + ik_z z)$ for the white and black nodes, respectively, where $\psi_{a,b}$ is the wavefunction. Then the corresponding Hamiltonian is

$$H(\mathbf{k}) = \sum_{i=0}^{3} d_i(\mathbf{k})\sigma_i, \tag{S6}$$

where $d_0 = (\kappa_z + \nu_z)\cos(k_z a)$, $d_1 = (\kappa_x + \nu_x)\cos(k_x a) + 2\kappa_y \cos(k_y a)$, $d_2 = (\kappa_x - \nu_x)\sin(k_x a)$, $d_3 = -\delta_C - (\kappa_z - \nu_z) + (\kappa_z - \nu_z)\cos(k_z a)$. We confirm that the Hamiltonian of this two-band system satisfies time-reversal symmetry (T) rather than parity (P) inversion since $TH(\mathbf{k})T^{-1} = H(-\mathbf{k})$ and $PH(\mathbf{k})P^{-1} \neq H(-\mathbf{k})$. After diagonalization, the eigenvalues (normalized eigenfrequencies) are

$$\varpi_\pm = d_0 \pm \sqrt{d_1^2 + d_2^2 + d_3^2}, \tag{S7}$$

which are related to the eigenfrequencies by Eq. (S5).

2. Realization of ideal type-II WPs

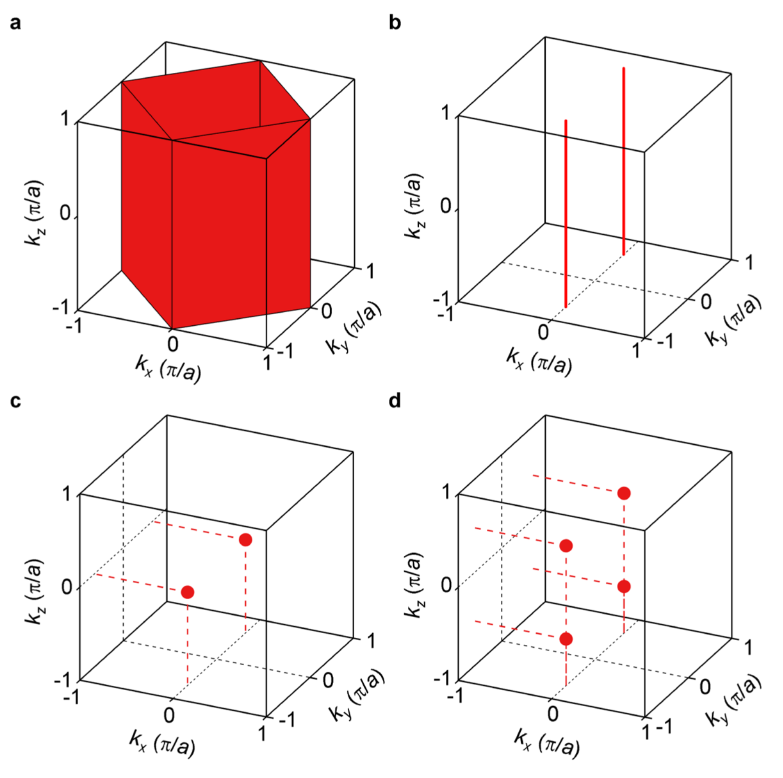

Fig. S1 Band degenerate points (BDPs) in momentum space where $C_a = C_b$ and $C_1 = C_2 = C_3 = C_4 = C_5$ in **a**, $C_a = C_b$, $C_1 \neq C_2$ and $C_4 = C_5$ in **b**, $C_a = C_b$, $C_1 \neq C_2$ and $C_4 \neq C_5$ in **c**, and $C_a \neq C_b$, $C_1 \neq C_2$ and $C_4 \neq C_5$ in **d**.

We consider a topological circuit as shown in Fig. 1**a** with $C_a = C_b$ and $C_1 = C_2 = C_3 = C_4 = C_5$. For an isolated layer in the x-y plane, there are two bands that are degenerate at the boundary of the square two-dimensional (2D) BZ and exhibit a quadratic degeneracy at the corners [6]. First, we stack identical layers along the z direction. In the Hamiltonian, both d_2 and d_3 are zero. The band degenerate points (BDPs) satisfy $\cos(k_x a) + \cos(k_y a) = 0$ and they are independent on k_z. The BDPs form a square tube with rotational axis along the k_z direction, as shown in Fig. S1**a**. Second, in order to break P of the circuit, we break the mirror symmetry in the x direction $M_x := x \rightarrow -x$ by setting $C_1 \neq C_2$, which leads to the splitting of the square degeneracy in the k_x-k_y plane [6,7]. In the three-dimensional (3D) BZ, the BDPs form a pair of lines along the k_z direction with $k_x = 0$ and k_y being determined by the sum of C_1 and C_2, and they have linear dispersion in the k_x and k_y directions. Here we set $C_1 + C_2 = C_3$, so that the

degenerate lines are projected to (0, ±2/3)π/a, as shown in Fig. S1**b**. Third, to isolate the BDPs, we need to break the line degeneracy. This is achieved by setting $C_4 \neq C_5$. In this case, there are two 3D degenerate points located at (0, ±2/3,0)π/a, as shown in Fig. S1**c**. However, these BDPs are not type-II WPs since they have quadratic dispersion in the k_z direction. Fourth, in order to move the BDPs to the positions that have linear dispersion, we use resonators with two different resonant frequencies by setting $C_a \neq C_b$. The frequency of the BDPs is related to the grounded capacitors C_a and C_b. By changing the grounded capacitors, we can move the position of the BDPs. Here a special relation $C_a - C_b = -2(C_4 - C_5)$ is employed. In this case, there are four degenerate points with linear dispersion located at (0, ±2/3, ±1/2)π/a in the first BZ, as shown in Fig. S1**d**. Besides, the capacitive coupling from the identical resonators in the z direction leads to a strongly tilted band structure in the k_x-k_z plane with $k_y = \pm 2\pi/3a$. These BDPs are exactly the ideal type-II WPs that we have realized.

3. Ideal type-II WPs in momentum space and Berry curvature

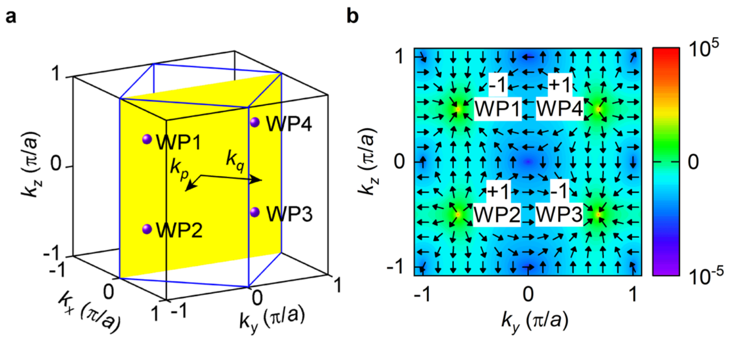

Fig. S2 **a,** Schematic of momentum space, showing the 3D BZ (blue lines) and the four ideal type-II WPs. **b,** Berry curvature in the $k_x = 0$ plane, where the arrows represent the direction of the vector field and the color scale indicates the logarithmically scaled magnitude. WP2 and WP4 act as sources of Berry curvature, and WP1 and WP3 act as sinks. Their Chern numbers are also labeled.

In momentum space, the positions of the four ideal type-II WPs are shown in Fig. S2**a**. Type-II WPs are always created or annihilated in pairs with opposite chirality and each

point acts as either a source or sink of Berry curvature. In Fig. S2**b**, we show the Berry curvature near the type-II WPs in the $k_x = 0$ plane, where the arrows represent the direction of the vector field and the color scale indicates the logarithmically scaled magnitude. We can see that type-II WPs are topological monopoles of a quantized Berry flux characterized by chirality, where WP2 and WP4 act as sources, and WP1 and WP3 act as sinks. Integrating the Berry curvature over a surface enclosing a type-II WP in momentum space [8], the calculated Chern numbers of the lower topological band for WP2 and WP4 are +1, and WP1 and WP3 are -1, as shown in Fig. S2**b**, which confirm their opposite chirality.

4. Experimental implementation of topological circuits

Figs. S3**a**-**b** show two types of unit circuit boards which contain the white and black resonator nodes, respectively. Two topological circuits shown in Figs. 2**a** and 3**a** are assembled using the unit circuit boards, where the periodic boundaries are realized by connecting the resonator nodes as a loop to avoid the finite size effect and the finite boundary is realized by grounding [9]. Figs. S3**c-d** show the diagram of entire measurement setup and site-resolved transmission measurement system, respectively.

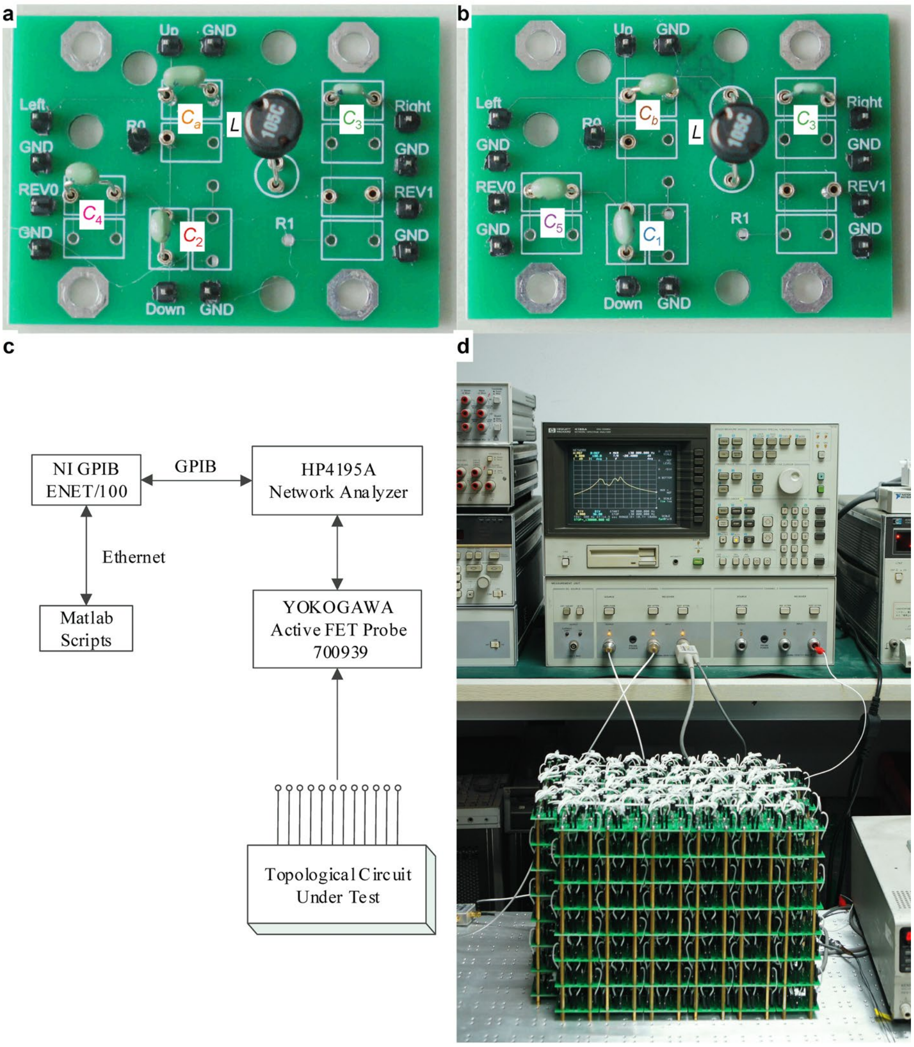

Fig. S3 **a-b**, Two types of unit circuit boards which contain the white and black resonator nodes, respectively. **c**, Diagram of the entire measurement setup. **d**, Site-resolved transmission measurement system.

5. **k·p** model

There are four ideal type-II WPs at (0, ±2/3, ±1/2)π/*a* in the first BZ. Around the type-II WPs, the circuit Hamiltonian can be reduced to the **k·p** model. By taking the type-II WP located at (0, 2/3, 1/2)π/*a* as an example, the reduced Hamiltonian is

$$H(\mathbf{k}) = \sum_{i=0}^{3} d_i(\mathbf{k})\sigma_i, \tag{S8}$$

where

$$
\begin{aligned}
d_0 &= -(\kappa_z + \nu_z)\left(k_z a - \frac{\pi}{2}\right), \\
d_1 &= -\sqrt{3}\kappa_y\left(k_y a - \frac{2\pi}{3}\right), \\
d_2 &= (\kappa_x - \nu_x)k_x a, \\
d_3 &= -(\kappa_z - \nu_z)\left(k_z a - \frac{\pi}{2}\right),
\end{aligned}
\tag{S9}
$$

and the normalized eigenfrequencies are

$$
\bar{\omega}_{\pm} = d_0 \pm \sqrt{d_1^2 + d_2^2 + d_3^2}. \tag{S10}
$$

According to Eq. S5, the frequency is linearly dependent on the normalized frequency near the type-II WPs. Thus, the band structures have linear dispersion. By setting k_x = 0 and $k_y = 2\pi/3a$, we find that the two normalized group velocities $\partial\bar{\omega}_{\pm}/\partial(k_z a)$ at the type-II WP are $v_{g,1} = -2\kappa_z$ and $v_{g,2} = -2\nu_z$, respectively.

6. Lossy effect in the topological circuit

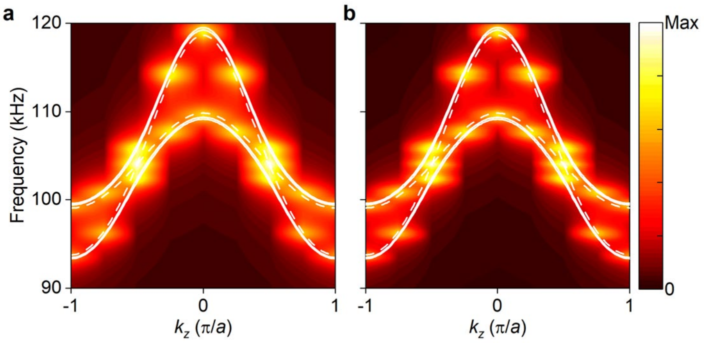

Fig. S4 Lossy effect in the topological circuit. The simulated band structures for the case in Fig. 3**d** under **a**, Q = 75 @100 kHz (R_s = 8.4 Ω) and **b**, Q = 120 @100 kHz (R_s = 5.2 Ω), respectively. The curves correspond to theoretical results.

The lossy effect of the topological circuit is mainly due to the serial resistance of inductors. If the inductors have a low Q factor (large serial resistance), resonant peaks in the transmission spectrum may merge together. Thus, to observe clear dispersion bands, it is critical to use high Q inductors. As an example, we do simulations for the case in Fig. 3**d** under Q = 75 @100 kHz (R_s = 8.4 Ω) and Q = 120 @100 kHz (R_s = 5.2

Ω), respectively. For simplicity, we neglect the dispersion of serial resistance. As shown in Fig. S4, the resolution of dispersion bands is improved by increasing the Q factor.

Following this guideline, it may be possible to further improve the resolution of experimental band structures by using customized inductors with ultrahigh Q factors. Several techniques can be employed during the material selection, structure design, and fabrication process. First, Litz can be used as the inductance coil to reduce the energy loss [10]. Second, Ni-Zn ferrite can be selected as the magnetic core of inductors [11]. Finally, the shape, and the number of wraps and layers of inductance coil can be optimized.

7. Group velocities of the surface states

The propagation directions of the surface states are determined by the tilting direction of type-II WPs [7]. For a fixed k_z, the group velocities of the surface states have the same sign. However, with a single frequency excitation in circuits, the surface states with both positive and negative k_z are excited. Therefore, bidirectional propagation of the surface states was experimentally observed, as shown in Fig. S5. Considering the periodic boundary condition in the q direction, the transmission distribution in the q direction is symmetric with respect to the input port.

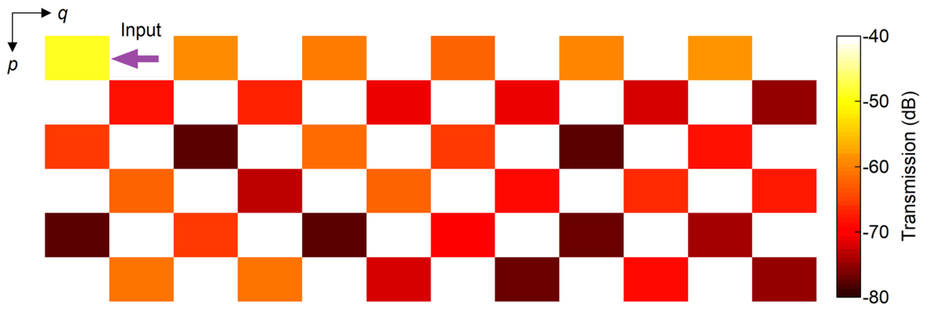

Fig. S5 Transmission distribution of the surface states in the whole p-q plane.

8. Fermi arcs

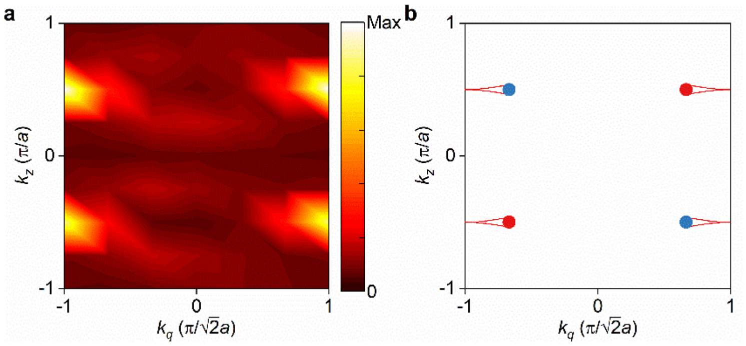

Fig. S6 Fermi arcs from the **a**, experimental measurement and **b**, theoretical results. The Fermi arcs connect the projections of two pairs of type-II WPs (red and blue dots in **b**).

The surface states are seen as Fermi arcs connecting the projections of type-II WPs with opposite chirality in the surface BZ. In order to demonstrate the Fermi arcs, we trace out the equifrequency contour of the surface states in the surface BZ (k_q-k_z plane) at 104.0 kHz. As shown in Fig. S6, the Fermi arcs at the band edges connect the projections of a pair of type-II WPs.

9. Adjustability of ideal type-II Weyl circuits

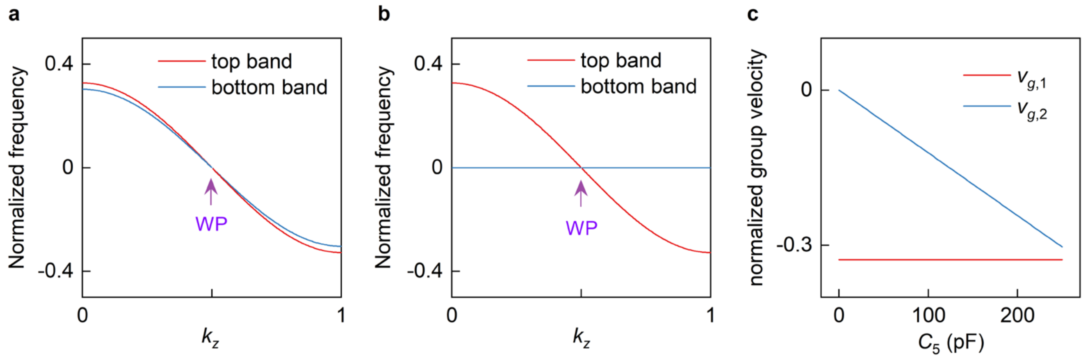

Fig. S7 Adjustability of group velocities. **a-b**, 1D band structures obtained by sweeping k_z at (k_x, k_y) = (0, 2/3) π/a with C_4 = 270 pF, where C_5 = 250 pF and 0 pF in **a** and **b**, respectively. The frequencies are normalized. The group velocities near a type-II WP at (0, 2/3, 1/2) π/a for the top and bottom bands are $v_{g,1}$ and $v_{g,2}$, respectively. In **a**, the two group velocities near a type-II WP are in the same direction and they have almost equal magnitudes. While in **b**, one group velocity $v_{g,2}$ changes to zero with a constant $v_{g,1}$, and a flat band appears. **c,** The dependence between the coupling capacitance in the z

direction C_5 and the normalized group velocities at the ideal type-II WP.

Here, we discuss the adjustability of our ideal type-II Weyl circuits. First, according to Eq. S2, the frequency of ideal type-II WPs is adjustable by using different inductors in the grounded LC resonators. This adjustability is challenging in other systems. Second, the group velocities near type-II WPs are easily adjustable due to the configurable circuit components. As an example, Fig. S5 shows the dependence between the coupling capacitance in the z direction and the group velocities near a type-II WP at (0, 2/3, 1/2)π/a. According to Supplementary Information section 5, the group velocities near the type-II WP for the top and bottom bands are $v_{g,1} = -2C_4/C$ and $v_{g,2} = -2C_5/C$, respectively, where C is the normalization capacitance, and C_4 and C_5 are the coupling capacitances that connect the white and black nodes in the z direction, respectively. As shown in Fig. S5**a**, when C_4 and C_5 are approximately equal, the top and bottom bands approach each other and $v_{g,1}$ nearly equals to $v_{g,2}$. It is worth to note that, the normalized frequency of WP does not change because the relation $C_a - C_b = -2(C_4 - C_5)$ still holds by adjusting C_a and C_b. However, as shown in Fig. S5**b**, when the coupling capacitance C_5 are disconnected directly, the group velocity $v_{g,2}$ changes to zero with a constant $v_{g,1}$, and the emergence of a flat band indicates the topological Lifshitz phase transition from type-II WPs to type-I WPs [12]. For clarity, Fig. S5**c** shows that the group velocity $v_{g,2}$ at the type-II WP changes in a wide range with the fixing of C_4 and adjusting of C_5. The above discussion implies that our topological circuits provide a clean and adjustable platform to observe ideal type-II WPs.

References

1. Soluyanov, A. A., Gresch, D., Wang, Z. et al. Type-II Weyl semimetals. Nature **527**, 495-498 (2015).
2. Jia, N., Owens, C., Sommer, A., Schuster, D. et al. Time- and Site-Resolved Dynamics in a Topological Circuit. Phys. Rev. X **5**, 021031 (2015).
3. Albert, V. V., Glazman, L. I. & Jiang, L. Topological Properties of Linear Circuit

Lattices. Phys. Rev. Lett. **114**, 173902 (2015).

4. Bao, J., Zou, D., Zhang, W. et al. Topoelectrical circuit octupole insulator with topologically protected corner states. Phys. Rev. B **100**, 201406 (2019).
5. Imhof, S., Berger, C., Bayer, F. et al. Topolectrical-circuit realization of topological corner modes. Nat. Phys. **14**, 925-929 (2018).
6. Chong, Y. D., Wen, X.-G. & Soljačić, M. Effective theory of quadratic degeneracies. Effective theory of quadratic degeneracies. Phys. Rev. B **77**, 235125 (2008).
7. Yang, Z. & Zhang, B. Acoustic Type-II Weyl Nodes from Stacking Dimerized Chains, Phys. Rev. Lett. **117**, 224301 (2016).
8. Bernevig, B. A. & Hughes, T. L. Topological insulators and topological superconductors (New Jersey, Princeton University Press, 2013).
9. Ashcroft, N. W. & Mermin, N. D. Solid State Physics (New York, Harcourt College Publishers, 1976).
10. Rahimi-Kian, A., Keyhani, A. & Powell, J. M. Minimum loss design of a 100 kHz inductor with litz wire, IAS '97. Conference Record of the 1997 IEEE Industry Applications Conference Thirty-Second IAS Annual Meeting, New Orleans, LA, USA, **2**, 1414-1420 (1997).
11. Lee, J.-J., Hong, Y-K., Bae, S. et al. High-Quality Factor Ni-Zn Ferrite Planar Inductor, IEEE Transactions on Magnetics **46**, 2417-2420 (2010).
12. Yang, Y., Gao, W., Xia L. et al. Spontaneous Emission and Resonant Scattering in Transition from Type I to Type II Photonic Weyl Systems. Phys. Rev. Lett. **123**, 033901 (2019).
